# Family meetings in palliative care: Multidisciplinary clinical practice guidelines

**DOI:** 10.1186/1472-684X-7-12

**Published:** 2008-08-19

**Authors:** Peter Hudson, Karen Quinn, Brendan O'Hanlon, Sanchia Aranda

**Affiliations:** 1 Centre for Palliative Care Education & Research, St Vincent's and The University of Melbourne, PO Box 2900, Fitzroy, Victoria 3065, Australia; 2 The Bouverie Centre, La Trobe University, 8 Gardiner Street, Brunswick, Victoria 3056, Australia; 3 School of Nursing and Social Work, The University of Melbourne, Level 5, 234 Queensberry Street, Carlton, Victoria 3010, Australia

## Abstract

**Background:**

Support for family carers is a core function of palliative care. Family meetings are commonly recommended as a useful way for health care professionals to convey information, discuss goals of care and plan care strategies with patients and family carers. Yet it seems there is insufficient research to demonstrate the utlility of family meetings or the best way to conduct them. This study sought to develop multidisciplinary clinical practice guidelines for conducting family meetings in the specialist palliative care setting based on available evidence and consensus based expert opinion.

**Methods:**

The guidelines were developed via the following methods: (1) A literature review; (2) Conceptual framework; (3) Refinement of the guidelines based on feedback from an expert panel and focus groups with multidisciplinary specialists from three palliative care units and three major teaching hospitals in Melbourne, Australia.

**Results:**

The literature review revealed that no comprehensive exploration of the conduct and utility of family meetings in the specialist palliative care setting has occurred. Preliminary clinical guidelines were developed by the research team, based on relevant literature and a conceptual framework informed by: single session therapy, principles of therapeutic communication and models of coping and family consultation. A multidisciplinary expert panel refined the content of the guidelines and the applicability of the guidelines was then assessed via two focus groups of multidisciplinary palliative care specialists. The complete version of the guidelines is presented.

**Conclusion:**

Family meetings provide an opportunity to enhance the quality of care provided to palliative care patients and their family carers. The clinical guidelines developed from this study offer a framework for preparing, conducting and evaluating family meetings. Future research and clinical implications are outlined.

## Background

Palliative care is expected to be holistic and multidisciplinary; it is provided to both the patient and their family [1]. Effective communication between the patient, the family and health care providers is integral to optimal palliative care. One method of facilitating communication is a family meeting, also referred to as a family conference [2]. Family meetings between the patient, their family and health care professionals are undertaken for multiple purposes including the sharing of information and concerns, clarifying the goals of care, discussing diagnosis, treatment, prognosis and developing a plan of care for the patient and family carers [2-4].

However despite the promotion of family meetings as an essential tool for information sharing and goal clarification in specialist palliative care settings, it has been reported that sparse evidence exists to demonstrate the process for training staff to conduct or participate in them [3]. It has also been claimed that there is a dearth of published literature describing when such meetings should be initiated, who should attend them, how they should be conducted and evaluated [5].

We set out to develop practice guidelines for conducting family meetings within the context of the specialist palliative care setting, based on the best available evidence and complemented by consensus based expert opinion. For the purposes of these guidelines we define a specialist palliative care setting as a health care environment or service that specifically focuses on the care of patients with an advanced incurable disease and their family. An evaluation of the effectiveness of these guidelines in clinical practice will be reported separately.

## Methods

Key questions guiding the development of the guidelines were: (a) How should family meetings be convened and structured? (b) What content is essential? and (c) Who should attend and lead them?

Development of the guidelines was based on the following three methods: (1) A literature search using MEDLINE (1995 – 2007), CINAHL (1995 – 2007), and PsycINFO (1995–2007) databases. The principal search terms used either singly or in combination, were: family, carers, case, conference, meeting, hospice and palliative care. Key palliative care textbooks (eg [6]) and their reference sections were also hand searched for relevant information; (2) The research team considered relevant models and theories to support the conceptual framework to underpin the guidelines; (3) (a) The research team prepared a draft version of the guidelines which was then reviewed by an expert panel alongside the conceptual framework. Members of the panel were purposively chosen (based on their experience in conducting family meetings) and invited by mail to participate; (b) The guidelines were further refined and their clinical applicability was assessed via two focus groups. The first (focus group 1) comprised multidisciplinary specialists from three palliative care units and the other (focus group 2) comprised multidisciplinary palliative care specialists from three large Melbourne hospitals. The inclusion criteria required that participants had experience in participating in family meetings. Letters of invitation were sent to the managers of each of the services requesting that potential participants contact the research team. Ethical approval was provided by St Vincent's Hospital Melbourne, Australia and written consent was obtained from all participants. The focus group questions centred on asking participants to briefly describe: current practice in relation to family meetings in their setting; key elements of family meetings and then to provide comment on the draft version of the clinical guidelines.

Data from the expert panel and focus groups were audiotaped and transcribed verbatim. These data were then content analysed in accordance with the structured questions set by the research team.

## Results

### Literature Review

The literature search revealed only three published articles in peer reviewed journals specifically related to family meetings in the specialist palliative care setting [2,3,7]. Due to the limited amount of palliative care specific literature; evidence from non-specialist palliative care settings, including intensive care and aged care was also reviewed and included for the purposes of the literature review.

One palliative care related article acknowledged that most health professionals do not receive sufficient training to conduct family meetings [3]. The authors reported on the evaluation of a training program focused on preparing medical and social work students for conducting family meetings in palliative care. However, the study was primarily about the outcomes of the educational initiative rather than specific details about how to convene and conduct family meetings or whether or not family meetings were beneficial [3]. One article was prepared as an educative tool for health professionals and provided recommendations for conducting family meetings in palliative care, seemingly based on expert opinion in the most part [7]. The final article was an informational tool about family meetings designed for patients and families [2], although the process for determining the content was not outlined. Recommendations for conducting family meetings were identified in some palliative care books, but these were also based on expert opinion (for example [8]).

The opinion based family meeting guidelines for health professionals advocated suitable planning and an overt purpose in order to conduct an effective meeting. Other key recommendations included giving consideration to who should attend, a meeting place that ensures privacy and a method for disseminating outcomes of the meeting.

Similar recommendations on how to conduct a family meeting exist in the aged care setting [9]. While these suggested approaches appear useful (as was the case for palliative care based suggestions), the basis for their content is not explicitly outlined and it is unclear as to whether their utility has been tested. One qualitative study was identified which aimed to explore the opinions of participants in family meetings in a geriatric rehabilitation hospital. Staff, patients and families participated in focus groups, completed surveys and individual semi-structured interviews [5]. Although high levels of satisfaction with family meetings were reported, patient interviews revealed a lack of informed consent and lack of clarity regarding the purpose of meetings. An unclear agenda was identified by staff, patients and families as the underlying reason for unsatisfactory meetings.

A practice model for nurses working with families has been developed [10]. It includes family interviews conducted over approximately an hour, as well as 15 minute interviews designed to be conducted within the course of normal nursing duties. The key elements of a family interview identified by these authors included: therapeutic conversations; manners; family genograms and ecomaps; therapeutic questions and commending family and individual strengths. However, there does not appear to be research evidence supporting the effectiveness of this model in practice.

The conduct of family meetings has also been described in the context of training medical practitioners [11]. Two related qualitative studies investigated the attitudes of residents (junior doctors) and their educators towards family conferences. A survey of 65 residents revealed that family meetings are: valued as a communication tool to provide medical information and reach consensus on care; occur primarily in inpatient settings in the context of crisis; fail to occur due to significant barriers; and are best taught experientially with the involvement of physician role models [11]. The accompanying survey of 12 teaching staff revealed major themes including: family conferences occur on a continuum of formality; experiential, in vivo training for family conferences is the primary training modality; specific skills are needed to conduct family conferences; and the barriers to conducting family conferences are formidable [11].

Our review identified several studies that explored family meetings within the context of the intensive care unit (ICU) [12-20]. Even though the ICU setting is considerably different from a typical palliative care unit, given the lack of research related to family meetings in the specialist palliative care setting we deemed it pertinent to highlight these studies. Furthermore, there are some similarities between the two care settings: a considerable proportion of the patients die in both ICU and palliative care units and patients are often too unwell to be involved in the family meeting.

A study involving qualitative analysis of 51 family conferences across four ICUs in the USA and a survey of 169 family members regarding their satisfaction with communication found that increasing the frequency of three types of clinicians' statements during family conferences was associated with increased family satisfaction[14]. These statements included (a) assurances that the patient will not be abandoned before death, (b) assurances that the patient will be comfortable and will not suffer and (c) support for family's decisions about end-of-life care, including support for family's decision to withdraw or not to withdraw life-support. A related study used the same data to explore missed opportunities for providing support and information to families [15]. The researchers determined that in 29% of family conferences opportunities were missed by the convenors of the meetings. These missed opportunities fell into three broad categories: listening and responding to family; acknowledging and addressing emotions; pursuing key principles of medical ethics and palliative care such as exploration of patient preferences, explanation of surrogate decision making and affirmation of non-abandonment. Another sub study, (drawing on similar data) explored shared decision making strategies in the ICU and concluded there was a small degree of empirical support for this approach for family meetings [19].

Lilly and colleagues examined the effectiveness of a proactive patient and family centred communication strategy within the ICU [20]. The intervention involved focused health professional meetings with the patient and/or their family within 72 hours of admission and incorporated a review of: the medical facts, the patient's perspective on impact of the illness/situation, and development of a care plan. Using a before- and- after study design, the intervention demonstrated reduction in the time spent in ICU and earlier withdrawal of advanced supportive technology.

A study conducted in France aimed to measure the amount of time physicians in ICU spent with patients' families by examining contact over a 24 hour period with 951 patients [17]. The median time spent providing information to families was 16 minutes per patient, with 20% of the time spent explaining the diagnosis, 20% on explaining treatments, and 60% on explaining the prognosis. One third of the time was spent listening to family members. Subsequent analysis revealed that the presence of more than one bed in the room was associated with less time spent on providing information, while a range of patient and family factors were associated with more time being spent on providing information. In an apparently related study, a randomised controlled trial demonstrated that family members who participated in a structured ICU family meeting format and received a brochure on bereavement, had less anxiety and depression than those receiving standard care[12].

A cross sectional study involving audiotaping family meetings in the ICU concluded that family satisfaction with communication was increased when they were given ample opportunity to voice their concerns, rather than just listening to the medical staff [16]. Lautrette and colleagues [18] claim from their review of ICU based family meeting literature that family meetings improve communication between relevant parties and help to alleviate families' emotional burden.

In summary, from our review of the literature, while family meetings are promoted as a common and valuable tool within the context of specialist palliative care there is limited evidence related to how they should be conducted and indeed whether they are valuable, most evidence being extrapolated from other settings. Furthermore, despite the apparent support for family meetings, there is limited evidence from the literature that explores ways of preparing or educating health professionals to conduct them [3]. While the findings from other clinical settings such as ICU help inform family meeting guideline development it would be unwise to generalise these results to the specialist palliative care setting given the different population (even though there are some similarities) and dissimilar foci of the meetings.

### Conceptual framework

The conceptual foundation for the family meeting guidelines for palliative care was derived initially by the research team from the available literature and then with input from the expert panel. Research team and panel members drew upon their knowledge of various conceptual and theoretical models which appeared to be applicable to underpinning the guidelines. The final conceptual foundation was informed by: (1) The transactional model of stress and coping, (2) Single session therapy, (3) Family consultation model and (4) Therapeutic communication principles. A brief overview of each is now provided.

#### Transactional model of stress and coping

The diversity of responses related to end-of-life issues from patient and family carers can be understood from a psychological perspective based on a transactional model of coping in which cognitive appraisals are made to determine the possible impact of a potentially stressful event [21-23]. The carer's perception of self-efficacy, for example, or the greater the number of resources at their disposal to manage an event, the more likely the individual will more favourably respond to the situation. Such resources include feelings of preparedness, competence, having sufficient social support, and adequate information. This model was utilised favourably in other interventions that have assisted family carers to feel more prepared and better informed about the role of supporting a dying relative [24,25]. Given that family meetings tend to involve information provision about the patient and family's current status, future care options and resources the transactional model provides a useful means of approaching families.

#### Single Session Therapy

The psychologist, Moshe Talmon is most often associated with the development of Single Session Therapy which he articulated in his texts in the early 1990s in the United States [26]. Talmon initially researched the number of sessions attended by individuals seeking treatment in a mental health outpatient setting; the most common number of sessions (or mode) attended was one. Although usually viewed as 'drop outs' or treatment failures, Talmon discovered on follow up of a group of these clients that three quarters of them reported that they were improved or much improved after one unplanned session. He and his colleagues also found that when this single session was planned, the proportion of those reporting benefit increased by approximately 10% [26]. As interest in Single Session Therapy increased, its application was extended to family therapy and other settings, particularly within Australia where the results were similarly encouraging. (See, for example [27-30]).

Over time the practice of Single Session Therapy has developed into an approach and set of techniques that emphasizes both the client and the therapist approaching their single session with an attitude of trying to make the most of each meeting (even if further sessions are needed). It also sees Single Session Therapy as part of a process that begins with initial contact and includes phone contact after the session with the client. An advantage of the single session method is that it has been used in combination with a range of counselling approaches with differing theoretical orientations.

More recently, there have been attempts to integrate Single Session approaches, including the types of questions used, into existing work roles; for example, as part of intake or assessment processes in mental health settings. In other contexts it has been used to inform meeting practices with families, where ongoing treatment and contact occurs with an individual but where professional contact with their family may be occasional or episodic. Family meetings in palliative care typically occur only once (usually as a consequence of limited time). The tenets of single session therapy therefore seem relevant to the development of family meetings clinical guidelines. Although we contend that conducting a routine family meeting should not be considered as 'therapy', some principles of single session therapy appear applicable.

#### Family Consultation

Family Consultation is a model of family intervention that emerged in the mental health field in the United States in the late 1980s [31]. It arose in response to a lack of responsiveness by services to the needs of families whose relatives were experiencing mental health problems. There was also a sense that these families were often 'co-opted' into particular forms of involvement deemed helpful to the client by professionals without any consideration of family members' own needs or wishes [32]. Typically, family consultations happen on a 'one off' or 'as needed' basis usually defined by the family. The consultations may be provided by professionals within the service setting where their family member is being treated or in separate family support services. The person with the mental health problem may or may not participate in the consultation. In contrast to family meetings in many settings, there is a conscious shift by the practitioner from an exclusive focus on how families support treatment of their ill relative to recognising and responding to the impact of the condition on other family members.

The model assumes a position of family competence and seeks to cultivate a respectful and reciprocal relationship between health professionals and families [33]. In practice the professional conducting a family consultation seeks to clarify how families want to be involved in various elements of the treatment process and to develop a plan to meet the expressed needs of family members. [34] Research in relation to family consultation is limited although in one study the use of this approach resulted in an increased sense of self-efficacy for family members in managing their relatives' mental illness [35]. Hence, given palliative care's remit to support the family alongside the patient, the family consultation model offers a useful framework to support the development of guidelines for conducting family meetings.

#### Therapeutic communication

Guidelines for therapeutic communication and psychosocial support for adults with cancer [36] offer strategies for eliciting and responding to cues from patients and family carers, which are highly relevant for palliative care related family meetings. Moreover, the guidelines highlight evidence from systematic reviews of randomised controlled trials with people with cancer that show that: (a) expressing empathy and listening actively improves psychological adjustment; (b) provision of comprehensive information about what to expect in the future promotes psychological well-being; and (c) an opportunity to discuss feelings with a health professional reduces psychosocial distress. Effective communication between health professionals and families is crucial to constructive family meeting outcomes. Consequently, the principles of therapeutic communication [36] were a core component of the guideline development.

### Refinement of the guidelines (expert panel and focus groups)

The research team developed the initial draft of the clinical guidelines based on their clinical experience, the aforementioned literature review and the conceptual framework. The guidelines were then refined by an expert panel comprising: family therapist, social worker, psychologist, clinical nurse consultant, pastoral care consultant, consumer representative, and a palliative care medical consultant.

After several iterations a penultimate version of the guidelines was presented at two focus groups for feedback to inform the final version of the guidelines. The first focus group consisted of multi-disciplinary team members from three metropolitan palliative care in-patient units. There were seven participants in this focus group. The health professionals represented the following disciplines: medicine (2); nursing (2); social work (1); occupational therapy (1); and pastoral care (1). The second focus group consisted of consultative palliative care service representatives from three major metropolitan hospitals. Eight participants contributed to this focus group including palliative care medical consultants (3); clinical nurses consultants (4); and social worker (1). Commensurate with the focus group questions (developed by the research team) the results were categorised in the following content areas: (a) description of current practice related to family meetings; (b) recommended core elements of family meetings and (c) feedback on the draft version of the family meeting clinical practice guidelines.

A summary of the results is now outlined. In relation to current practice regarding family meetings it was reported that:

• Meetings were typically offered based on need and not routinely;

• Meetings were typically convened and chaired by a social worker;

• The treating team usually determined who attended the meeting;

• Formal training in conducting family meetings does not occur as part of general training;

• Some units use a family meeting guide that they had developed internally;

• The typical purpose of meetings was to find out what a family already knows about patient's prognosis and whether there are any gaps in knowledge;

• Relevant health professionals generally try to meet before the meeting to clarify the goal of the meeting;

• The social worker (who is commonly the chairperson) usually documents the outcomes of the meeting in the patient' medical record;

• Documentation of the meeting discussion and outcome is not usually provided to the family;

• Meetings are not formally evaluated as part of a quality improvement strategies.

In relation to recommendations regarding core elements of family meetings it was reported that:

• Good communication skills were considered essential;

• A friendly and comfortable environment should be promoted;

• Common goals and objectives should be established for the meeting;

• Only key health professionals should be present at the meeting;

• A designated chairperson is required for the meeting;

• An agenda, specified aims and objectives should be confirmed at the start of the meeting;

• The meeting should also be used as a forum to provide and gather information;

• Allowing time at the beginning of the family meeting for introductions was considered essential.

In relation to feedback regarding the draft version of the clinical guidelines for family meetings there was general consensus that the guidelines were applicable to the clinical setting and contained the aforementioned key elements. However the following points were considered important to be included in the guidelines:

• Clarification of some terms throughout the protocol to reduce ambiguity was recommended;

• Limiting the number to 'one or two' family members and/or friends was highlighted as a potential challenge in practice;

• Ensuring that each discipline represented at the meeting has an opportunity to contribute was considered important;

• Where pertinent and if possible (resources permitting), offer a family meeting via teleconference.

### Clinical practice guidelines for conducting family meetings

The guidelines for conducting family meetings are outlined in Table 1. The following principles for conducting family meetings were also developed alongside the guidelines preparation process.

**Table 1:** Clinical Practice Guidelines for Conducting Family Meetings in Palliative Care

**1. Preparing for a family meeting**
**a) **On admission to the palliative care unit the relevant health professional should introduce the purpose of a family meeting and offer a family meeting to all lucid patients. This discussion should incorporate the role that palliative care has in supporting families as well as the patient.
**b) **Ask the patient to confirm one or two key family carers and/or friends who they approve to be involved in medical and care planning discussions. Note this in the medical record.
**c) **Conduct a family genogram to determine key relationships within the patient's family. It could be introduced thus: "Can I spend a few minutes just working out who is in your family?"
**d) **Seek the patient's permission to arrange a family meeting and ask if they have any particular issues/concerns or questions they would like discussed at the meeting. If the patient does not want to attend, seek their permission to conduct a meeting with key family and/or friends (as above). If the patient is unable to make an informed decision, offer the meeting to the next of kin or key family/friends who have been identified to receive information and care planning decisions related to the patient. Note: Where a patient has no family or appropriate proxy a legal guardian may need to be appointed.
**e) **Identify the most appropriately skilled person from the multidisciplinary team to convene the family meeting. This person will take responsibility for scheduling, invitations and coordination. Ideally this person should also act as the primary contact point for the key family carer(s).
**f) **Contact the primary family carer(s): provide an overview of purpose of the family meeting; offer to convene a meeting at a mutually acceptable time. Advise the carer that the meeting time will be confirmed in due course (i.e., once other attendees are arranged). Where pertinent, and if resources allow offer to conduct the meeting via teleconference. Establish the main questions and issues that the family carer would like discussed (refer Table 3). If the patient is participating in the meeting ask him/her to identify their key concerns. Note: If significant family conflict (or other major issue) is identified consider referral to a practitioner who is trained to work with complex issues within families (e.g. family therapist or health psychologist).
**g) **Determine which health care professionals should attend the family meeting. Invite key health care professionals based on the identified needs of the patient and family carer. The number of staff should be restricted, inviting only the relevant health professionals, so that the patient and family/friends do not feel overwhelmed. Note: Include a professional interpreter if required.
**h) **Confirm the family meeting time and location. Inform attendees of the scheduled start and finish time for the meeting. A comfortable room free of interruptions (including pagers and phones), tissues made available and conducive seating arrangements is recommended.
**2. Conducting a family meeting**
**a) **Introduction
Chairperson to:
**i) **Thank everyone for attending and introduce him/herself and invite others to introduce themselves and state their role.
**ii) **Establish ground rules in a non patronsing way e.g. *"We would like to hear from all of you, however if possible could one person please speak at a time, each person will have a chance to ask questions and express views." *Request no interruptions such as phones etc.
**iii) **Indicate the duration of meeting (recommended maximum time of 60 minutes).
**b) **Determine the understanding of the purpose of the family meeting.
Chairperson to:
**i) **Briefly **outline **the broad purpose of the family meeting (based on previous steps), and then confirm with the family and patient that their interpretation of the purpose of the meeting concurs.
For example:
"*We arranged this meeting to consider discharge planning options. Is this your understanding of the purpose of the meeting?" (If not reframe the meeting's purpose)*
*or*
*"From the things you mentioned on the questionnaire what is the most important thing you would like to discuss?"*
*or*
*"How could we be most helpful to you today?"*
**ii) **Ask the patient/family if there are any additional key concerns, and if pertinent, prioritise these and confirm which ones will be attempted to be dealt with at this meeting (others can be discussed at a future meeting or can perhaps dealt with on a one on one basis).
**iii) **Clarify if specific decisions need to be made (e.g. if the patient is to go home or not).
**c) **Determine what the patient and family already know. Possible questions may include,
"*What have you been told about palliative care*" as a way of clarifying, confirming etc.
"*Tell me your understanding of the current medical condition or current situation*?"
If pertinent provide information (in accordance with desire) on the patient's current status, prognosis and treatment options.
Ask each family member in turn if they have any questions about current status, plan and prognosis. Helpful questions may include, "*Do you have questions or concerns about the treatment or care plan?"*
For family discussion with non-competent patient (i.e. cognitively impaired or imminently dying).
Ask each family member in turn:
"*What do you believe your relative/friend would choose if they could speak for himself/herself?"*
*"In the light of that knowledge, what do you think should be done?"*
**d) **Address specific objectives of the meeting (as previously determined).
**e) **'Check in' periodically throughout with the patient and family carer to see if the discussion seems to be valuable and is in keeping with their needs e.g. *"Are we on track?"; "Is this what you wanted from today's meeting?"; "What haven't we touched on that's important to you?"*
Also consider taking a short break during the meeting (to give participants time to digest information) and then allow some time to refocus.
**f) **Offer relevant written or audiovisual resources. Examples include guidebooks, brochures, enduring power of attorney documents, advance care directive information and so forth.
**g) **Identify other resources, including possible referral to other members of the multidisciplinary team. Suggest scheduling a follow-up meeting if pertinent.
**h) **Concluding the discussion.
Summarize any areas of consensus, disagreements, decisions and the ongoing plan (i.e. clarify next steps) and seek endorsement from attendees (e.g. *"Are we all clear on the next steps?")*
Emphasize positive outcomes arising from the meeting.
Offer final opportunity for questions, concerns, or comments. E.g. *"What hasn't been covered today that you would have like to discuss?" *or *"Are there any questions you had that haven't been answered yet?"*
Remind patient and family carers to review the recommended written resources.
Identify one family spokesperson for ongoing communication.
Thank everyone for attending.
**3. Documentation and follow-up**
**a) **Document who was present, what decisions were made, what the follow-up plan is and share this with the care team (see Table 4).
**b) **Offer the patient/family a copy of the main content of the meeting and file a copy of this document in the patient's medical record.
**c) **Liaise with the primary family carer within a few days after the meeting to determine if the meeting was helpful (see Table 5)
**d) **Maintain contact with the key family spokesperson, including attending scheduled follow-up meetings or telephone calls as needed.

#### Guiding principles for conducting family meetings

• Family meetings can be a useful way to assist patients and family members to clarify goals of care, consider site of care options, and to share information. Ideally they provide a safe environment where issues and questions can be raised and appropriate strategies agreed upon.

• Strategies to support family carers are a core component of palliative care; hence service providers have a responsibility to *offer *family meetings based on need.

• Service providers should view family meetings as mutually beneficial. They are not only potentially valuable for patients and family carers; they may also provide a resource effective way to explain what the service can and cannot offer. Such meetings provide an opportunity to triage priority issues and a way to make referrals to other health professionals or other institutions early in the care planning phase.

• Family meetings should *not *be used as an opportunity for health care professionals to debate a patient's medical status; in this situation, a case conference should be convened prior to the family meeting.

• Family meetings should not be saved for 'crisis' situations. Instead, a preventative approach is advocated where issues are anticipated before they become major dilemmas. Hence a proactive rather than reactive approach to care is fostered.

• Ideally, family meetings are *offered *routinely on admission, and conducted at a pertinent time thereafter.

• Facilitators of family meetings require appropriate skills in group work, therapeutic communication and palliative care. We contend that the decision about who (i.e. which discipline) should convene and facilitate a family meeting is best determined on pragmatic grounds (local, site specific reasons) and not based on hierarchical reasons (i.e. based on authority). Hence the multidisciplinary team should determine who conducts the family meeting and presumably this may change depending upon skills, knowledge of the family and resources.

• Occasionally, family members may want to withhold details of the patient's prognosis from the patient; there may be incongruent wishes about the site of care; 'desire to die' statements may have been made by the patient; or there may be conflict within the family or difficulties regarding the transition from curative treatment to palliative care. In these circumstances we recommend the key resources and references to support therapeutic communication outlined in Table 2. Additionally, if it is known in advance that there is significant conflict (or other major issues) within the family, involving a family therapist or health psychologist may be appropriate.

**Table 2:** Recommended key references and resources for conducting family meetings

• Clinical practice guidelines for the psychosocial care of adults with cancer [36]
• Key communication skills and how to acquire them [37,38]
• Clinical practice guidelines for communicating prognosis and end-of-life issues with adults in the advanced stages of a life-limiting illness, and their carers [39]
• Responding to desire to die statements from patients with advanced disease: recommendations for health professionals [40]
• Supporting a person who requires palliative care: A guide for family and friends [41] (via: )
• 'Would you like to talk about your future treatment options?' discussing the transition from curative cancer treatment to palliative care [42]

Note: Publishers please aware that when inserting this box in the body of the text the ordering of the citations and reference list will need to be adjusted accordingly

• Pre-planning for the actual meeting is imperative as outlined in Table 3; so is comprehensive follow up after the meeting, as outlined in Tables 4 and 5.

**Table 3:** Pre-Family Meeting Primary Family Carer Questionnaire

Nb Conducted by phone [] or face to face [] by Family meeting convenor ............ [insert name]	
Now that I have explained about the family meeting and you have agreed to attend it would be useful for us if we had some more information in order to prepare for the family meeting.	
What are the main issues for you at the moment?	
(a) Greatest concern:	
................................................................................................................................................................	
(b) Second greatest concern:	
................................................................................................................................................................	
How upset/worried are you about these concerns? *(Place a cross on the line)*	
--------------------------------------------------------------------------------------------------------------------------------------------------------------------------------------------------------------------	
*(1) Not at all*	*As worried as I could possibly be (10)*
How often do these concerns arise? *(Place a cross on the line)*	
--------------------------------------------------------------------------------------------------------------------------------------------------------------------------------------------------------------------	
*(1) Not at all*	*All the time (10)*
Are there other difficulties you are coping with now? Please outline below:	
...............................................................................................................................................................	
...............................................................................................................................................................	
How much is the problem (or problems) interfering in your life? *(Place a cross on the line)*	
--------------------------------------------------------------------------------------------------------------------------------------------------------------------------------------------------------------------	
*(1) Not at all*	*Dominating my life completely (10)*
How confident do you feel in dealing with the problem(s)? *(Place a cross on the line)*	
--------------------------------------------------------------------------------------------------------------------------------------------------------------------------------------------------------------------	
*(1) Not at all*	*Extremely (10)*
What questions would you like to ask at the family meeting?	
...............................................................................................................................................................	
If you think of other questions between now and the family meeting, please write them down and bring them with you to the meeting.	
*Adapted with permission from Single Session Therapy Resource Guide (The Bouverie Centre 2006)*	

**Table 4:** Outcome of the Family Meeting

Below are key points to be recorded at the completion of the family meeting by the Family Meeting's Facilitator.				
A copy should be provided to the patient and family carer and one copy kept in the medical record.				
**Date of meeting: **__________________________				
**Name of family meeting facilitator: **_________________________________				
**Proposed purpose of the meeting: **__________________________________				
**FAMILY MEMBERS PRESENT**				
Name:		Relationship:		
Name:		Relationship:		
Name:		Relationship:		
**STAFF MEMBERS PRESENT**				
Name:		Role/Discipline:		
Name:		Role/Discipline:		
Name:		Role/Discipline:		
**KEY ISSUES RAISED AT THE MEETING**				
_________________________________________________________________________________				
_________________________________________________________________________________				
**KEY ACTIONS FROM THE MEETING**				
Current Situation	Goal	Action	Key Person to follow up	Review Date
				
				
				
*Adapted (with permission) from Single Session Therapy Resource Guide (The Bouverie Centre 2006)*				

**Table 5:** Post-Family Meeting Primary Family Carer Questionnaire

Nb Conducted by phone [] or face to face []. Completed by ............ [insert name]			
As a follow up to the recent family meeting we are interested in finding out how things are for you at the moment. Before the family meeting			
You nominated:			
.................................................................................................................................................................			
as the main problem to be discussed at the family meeting, and			
.................................................................................................................................................................			
as your second greatest problem.			
How upset/worried are you about this problem (or these problems) at the present time? *(Place a cross on the line)*			
----------------------------------------------------------------------------------------------------------------------------------------------------------------------------------------------------------------------			
*(1) Not at all*		*As worried as I could possibly be (10)*	
How often do these problems happen? *(Place a cross on the line)*			
----------------------------------------------------------------------------------------------------------------------------------------------------------------------------------------------------------------------			
*(1) Not at all*		*All the time (10)*	
How much is the problem (or problems) interfering in your life? *(Place a cross on the line)*			
----------------------------------------------------------------------------------------------------------------------------------------------------------------------------------------------------------------------			
*(1) Not at all*		*Dominating my life completely (10)*	
In what ways?...........................................................................................................................................			
How confident do you feel in dealing with the problem(s)? *(Place a cross on the line)*			
----------------------------------------------------------------------------------------------------------------------------------------------------------------------------------------------------------------------			
*(1) Not at all*		*Extremely (10)*	
You nominated the following questions as those you would like addressed in the family meeting:			
.................................................................................................................................................................			
To what extent do you feel these questions were addressed?			
.................................................................................................................................................................			
*Office use only:*			
	Pre-session	Post-session	Difference
How upset/worried:	........................	........................	........................
Problem frequency:	........................	........................	........................
Life interference:	........................	........................	........................
Confidence:	........................	........................	........................

• Suitable resources should be available to patients and family members who attend the meeting in order to complement the verbal information (e.g brochures about services available, carer guidebooks, treatment and drug information, etc).

## Discussion and Conclusion

Family meetings provide an ideal avenue to inform, deliberate, clarify and set goals for future care, based on discussions between health professionals and the patient and family. While there is consensus in the literature that family meetings are necessary and valuable, our study identified an absence of empirical evidence, within the context of specialist palliative care settings, to guide when they are required, who should attend, how they should be conducted and whether or not they are beneficial. Evidence from the intensive care context appears to offer the best guidance to date. While these data are important, caution is needed when considering their applicability to specialist palliative care settings where the population is somewhat different.

The feedback from clinicians involved in the focus groups in this study also complemented other claims that most palliative care health professionals receive little or no preparation to conduct family meetings [3]. Focus group participants also highlighted variations in current practice with regard to how family meetings were facilitated and by whom.

The clinical guidelines described herewith (based on a multidisciplinary research based approach) aim to assist health care professionals working in specialist palliative care to convene and conduct family meetings. Nonetheless, our study had several limitations and consequently more research is required. The clinical guidelines presented were in the most part based on expert opinion owing to the paucity of available research evidence. Accordingly, a formal evaluation of the benefits (or otherwise) of family meetings conducted using the guidelines is required. Furthermore, it would be advantageous to know whether the guidelines are applicable to other palliative care settings such as home care and aged care. Moreover, testing the utility of the guidelines in other countries is warranted. Our literature review included studies undertaken after 1995, hence there may have been evaluations of family meetings in palliative care conducted prior to this period. However, if so, the relevance of research findings almost 15 years on may be questionable. Evaluating strategies to prepare health care professionals to conduct family meetings would also be valuable [3].

The essence of palliative care provision is effective communication amongst health professionals, patients and their family carers. Family meetings are one potential method of interaction that may facilitate optimal care planning and support and seem to be commonly used in palliative care. To our surprise however, according to our review, there have been no research studies within specialist palliative care settings that have examined: when meetings should be convened, how they should be conducted; who should attend and whether or not they are effective. It is intended that the clinical guidelines presented here will aid health care professionals to plan, conduct and evaluate family meetings. However, we recommend that the utility of these guidelines undergo additional examination.

## Competing interests

The authors declare that they have no competing interests.

## Authors' contributions

PH was the principal investigator for the study and was awarded a Postdoctoral Research Grant to undertake the project. He oversaw the grant application, management of the research project and was responsible for drafting the manuscript and preparing the final submission and responsible for coordinating feedback regarding reviewer and editorial queries. KQ was a chief investigator for the project and assisted with design, data collection, project oversight and provided input into the manuscript focusing on the literature review. BO'H was a consultant for the project focusing on the areas of single session therapy and assisted primarily with the conceptual framework and literature review sections of the manuscript. SA was a chief investigator for the project and helped conceptualise the study design and provided feedback on draft versions of the manuscript. All authors read and approved the final manuscript.

## Pre-publication history

The pre-publication history for this paper can be accessed here:


